# Transcriptomic analysis revealed distinct transcriptional responses in *N. benthamiana* upon infiltration with different *Agrobacterium* strains

**DOI:** 10.3389/fpls.2026.1868449

**Published:** 2026-07-08

**Authors:** Tairu Wu, Weisong Pan, Waichin Li, Yuhan Wang, Xiwen Chen, Chuan Wu

**Affiliations:** 1 College of Metallurgy and Environment, Central South University, Changsha, China; 2 College of Biological Science and Technology, Hunan Agricultural University, Changsha, China; 3 Hunan Novomore Biotechnology Co., Ltd., Changsha, China; 4 Department of Science and Environmental Studies, The Education University of Hong Kong, Administrative Region, Hong Kong, Hong Kong SAR, China

**Keywords:** different *Agrobacterium* strains, infiltration, *N. benthamiana*, recombinant protein, transcriptome

## Abstract

Transient plant expression, which relies on *Agrobacterium* carrying T-DNA with target genes infiltrated into leaves for transient nuclear expression, is crucial for gene function research and the rapid production of pharmaceutical proteins. Commonly used laboratory *Agrobacterium* strains differ in their genetic backgrounds and modified helper Ti plasmids, which may induce varying degrees and durations of host immune responses and transcriptional reprogramming. However, the differential responses of *N. benthamiana*, the most commonly used host, to these strains remain unclear. In this study, we for the first time systematically investigated the transcriptional defense responses of *N. benthamiana* to six common laboratory *Agrobacterium* strains (*Agrobacterium tumefaciens* C58C1, GV3101, LBA4404, AGL-1, EHA105, and the *Rhizobium rhizogene* strain ArA4). Transcriptome profiling of tobacco leaves was conducted at 3 and 7 dpi to clarify the conserved and divergent regulation of tobacco immune pathways and stress responses by different strains. Additionally, we selected genes significantly upregulated in all strain-infiltrated groups and verified three novel defence-related genes, *PAR1*, *WRKY81*, and *DMR6*, that negatively regulate recombinant protein expression efficiency. We also analysed the expression patterns of previously reported immune-related genes that regulate recombinant protein yields, revealing the differential regulatory characteristics of tobacco in response to infiltration by different *Agrobacterium* strains. The results show that different strains have divergent activation patterns of immune genes and may adopt distinct colonization modes. We provide an important basis for selecting *Agrobacterium* strains for transient expression in *N. benthamiana*, optimizing tobacco transient expression systems, and constructing high-efficiency plant bioreactors.

## Introduction

*Agrobacteria* are a group of soil bacteria that can cause crown gall and hairy root diseases in a wide range of dicot plant species, such as *Agrobacterium tumefaciens*. Infected plants, whose T-DNA region is integrated, can produce opine in their proliferating tissues. *Agrobacterium* utilizes opine metabolism genes to break down opine into amino acids and sugars, which are then used by *Agrobacterium* as nitrogen and carbon sources (Zupan et al., 2000). Agrobacteria were mostly classified into three major biovars—BV1 (biovar 1), BV2 and BV3. BV1 includes a series of *Agrobacterium* spp. subdivided into several genomospecies, e.g., genomospecies 1 = G1. The well-studied reference strain C58 belongs to G8. BV2 is now designated as *Rhizobium rhizogenes*. *Rhizobium rhizogenes* has been of interest because of its ability to induce hairy root formation in plants. BV3 was reclassified as *Allorhizobium vitis* and *Allorhizobium ampelinum* (Weisberg et al., 2023).

A defining characteristic of agrobacteria is their ability to harbour oncogenic plasmids. Agrobacterial phytopathogenicity depends on either tumour-inducing (pTi) or root-inducing (pRi) oncogenic plasmids, which are mobilizable and can be horizontally transferred, acquired, or lost by bacterial cells (de Lajudie et al., 2019). These natural plasmids provide the basis for vectors to construct transgenic plants, which are approximately 200 kbp in size. *Agrobacterium* transfers its T-DNA into the nuclear genome of the host plant. T-DNA resides on pTi or pRi plasmids delimited by 25 bp imperfect repeats known as the right and left borders (RB and LB, respectively). The remaining regions of these plasmids contain gene clusters for DNA replication, virulence, opine utilization, and conjugation. The virulence (vir) region, which contains genes encoding type IV secretion system (T4SS) components, is responsible for the delivery of T-DNA to the nucleus of host plant cells (Sheng and Citovsky, 1996; Zupan et al., 2000). Agrobacterial infection and transformation are orchestrated by a cascade of chromosomal virulence (chv) and plasmid-encoded virulence (vir) genes. *Agrobacterium* initiates infection by attaching to plant surfaces via ChvA, ChvB, and cel proteins, which mediate exopolysaccharide and cellulose synthesis for stable adhesion. Plant-derived phenolic signals, sugars, and acidic pH are sensed by the two-component systems VirA/VirG and ChvG/ChvI, triggering the expression of virulence genes. The VirD1/D2 endonuclease-helicase complex nicks the T-DNA borders, generating a single-stranded T-DNA molecule covalently bound to VirD2. This complex is recruited to the T4SS by VirC1/C2 and VBP1–3. The T4SS, composed of VirB1–11 and VirD4, translocates the T-DNA and effector proteins including VirE2, VirE3, VirF, and VirD5 into host cells. Inside the plant, VirE2 protects the T-DNA from degradation and facilitates nuclear import, while VirD2 guides nuclear targeting and integration into the host genome. Additional effectors such as VirF and VirE3 suppress plant immunity, promoting successful transformation. Together, these plasmid-encoded virulence factors and chromosomal virulence (chv) genes coordinate the entire process of host recognition, T-DNA processing, secretion, nuclear delivery, and plant defense suppression (Weisberg et al., 2023). The two main components for successful *Agrobacterium*-mediated gene transfer, the T-DNA and the vir region, can reside on separate plasmids. These constitute the basis of modern binary Ti vector system (Hoekema et al., 1983). The function of the vir gene is provided by the disarmed Ti plasmid with a deleted T-DNA region residing in the *Agrobacterium* strain. “Disarmed” strains are avirulent strains due to the removal of oncogenes. The vector bearing the target gene within its T-DNA region is referred to as a binary vector.

C58 and Ach5 are the most important backgrounds used in biotechnology and belong to different phylogenic clades, namely, BV1-G1 and BV1-G8. In 2001, the first agrobacterial genome sequence was published for strain C58 (Goodner et al., 2001). To date, C58 is the most widely used strain in plant biotechnology with a relatively clear chromosomal background. Therefore, the C58 strain was used as the progenitor strain to modify several commonly used laboratory strains for conducting *Agrobacterium*-mediated transformation experiments. The most commonly used C58-derived strains are C58C1, which carry the disarmed pTiB6S3ΔT-DNA, and the helper plasmid pCH32. Four of the most frequently used strains, namely, GV3101, EHA101, EHA105, and AGL-1, are also C58 derivatives. GV3101 carried the disarmed pTipMP90 (pTiC58ΔT-DNA), and EHA105 and AGL-1 carried pTiBo542ΔT-DNA (Koncz and Schell, 1986; Lazo et al., 1991; Hood et al., 1993; De Saeger et al., 2021). In addition to C58, another well-studied strain, Ach5, harbours pTiAch5. Ach5 has also been previously modified for plant transformation, from which the disarmed laboratory strain LBA4404 was derived (Ooms et al., 1981; Hoekema et al., 1983).

*Agrobacterium* strains have long been widely utilized in related research and applications because of their unique ability to deliver genetic material into plant cells. This core capability mainly functions through two pathways: one is to cultivate stable transgenic plants, and the other is to achieve transient gene expression in plants. During the process of constructing stable transgenic plants, the target gene carried by the T-DNA region on the binary vector is first transferred from the *Agrobacterium* cells into the recipient plant cells, after which integration with the plant genome is completed. Researchers subsequently need to conduct specific screening steps to cultivate and regenerate successfully integrated cells, ultimately obtaining transgenic plants with stable genetic traits (Hwang et al., 2017). Compared with stable transformation, transient expression takes less time. Moreover, in research on plant gene function analysis, not all scenarios require the generation of stable transgenic plants. The core feature of transient expression is that only the gene encoding the target protein is transferred to the plant cells, enabling this gene to achieve short-term and high-level expression within the cells without being integrated into the plant genome. This technology not only has significant advantages, such as a short operation cycle, high target protein expression level, and low cost, but also effectively compensates for the shortcomings of stable expression technology in terms of cycle and transformation efficiency. Therefore, it has great application potential and unique advantages in multiple fields, such as molecular agriculture and biopharmaceuticals (Krenek et al., 2015).

A plant-based biofactory is a mature production system with advantages such as high cost-effectiveness, strong scalability, fast production speed, the ability to perform post-translational modifications, and the absence of harmful pathogen contamination. Several plant-derived vaccines have been produced, with some advancing to clinical trials (Ward et al., 2020, 2021). These diverse applications have driven efforts within the scientific community to enhance and optimize transient expression efficiency. However, agroinfiltration-mediated transient expression in *Nicotiana benthamiana* typically yields recombinant proteins at the milligram per gram fresh weight level, while low and unstable production remains a major industrial bottleneck (Akhtar et al., 2026). A recent study revealed that the *Rhizobium rhizogene* strain ArA4 also showed excellent expression efficiency in a transient expression system of *N. benthamiana*. Moreover, the expression efficiency of the *Agrobacterium* strain ArA4 even exceeded that of commonly used strains in the laboratory, such as GV3101 and C58C1 (Lopez-Agudelo et al., 2025). However, the differential responses of *N. benthamiana* to distinct *Agrobacterium* strains are currently unclear. Therefore, in this study, we used six different strains of *Agrobacterium* commonly used in the laboratory to infiltrate tobacco and characterized the transcriptional responses during the infection process of different *Agrobacterium* strains. The commonalities and differences exhibited by tobacco after infection with different *Agrobacterium* strains were compared.

The host immune response triggered by *Agrobacterium* infection often acts as a major barrier that restricts foreign gene expression (Dodds et al., 2023; Kopertekh, 2024). Recognition of pathogen-associated molecular patterns (PAMPs) from *Agrobacterium* activates a series of plant immune signalling pathways, including salicylic acid (SA) and jasmonic acid (JA) pathways, which further induce the expression of downstream defense-related genes. These immune genes not only enhance plant resistance to microbial invasion but also negatively regulate the transcription and accumulation of exogenous proteins by suppressing viral vector replication and triggering post-transcriptional gene silencing (Matsuo, 2022; Fang et al., 2026). Multiple defense-related genes in *Nicotiana benthamiana* negatively regulate recombinant protein production during *Agrobacterium*-mediated transient expression. NbCORE acts as a cell-surface receptor that recognizes bacterial cold shock protein (CSP) and activates pattern-triggered immunity; silencing *NbCORE* markedly weakens host defense and strongly enhances transient protein expression, particularly in older plants (Dodds et al., 2023). NbCERK1 functions as a key receptor for sensing chitin and peptidoglycan, while NbICS1, NbNPR1, and NbEIN2 are critical components of salicylic acid (SA) and ethylene signaling pathways. Suppression of these genes significantly attenuates plant immune responses and increases heterologous protein accumulation (Dodds et al., 2025). NbSABP2 and NbCOI1 participate in SA and jasmonic acid (JA) signaling pathways, respectively, and their silencing relieves immune inhibition to improve recombinant protein yields (Kopertekh, 2024). Moreover, NbGRP encodes a cell-wall–associated protein that restricts viral vector movement; knockout of *NbGRP* enhances viral infection efficiency and significantly boosts recombinant protein production (Fang et al., 2026). We selected several genes whose expression was significantly upregulated in the transcriptomes of tobacco leaves infiltrated with each of these six *Agrobacterium* strains and verified that they might affect recombinant protein expression. Three defence-related genes negatively regulate recombinant protein expression. Furthermore, we analysed the expression patterns of previously reported immune regulators that negatively regulate recombinant proteins in tobacco after infiltration with different *Agrobacterium* strains, revealing the differences in the transcriptional responses of tobacco to infection by different *Agrobacterium* strains. This provides an important basis for the selection of *Agrobacterium* for transient expression in *N. benthamiana*, the optimization of the tobacco transient expression system, and the construction of efficient plant bioreactors.

## Results

### Transcriptional profiles of *N. benthamiana* after infection with different *Agrobacterium* strains

**Figure 1:**
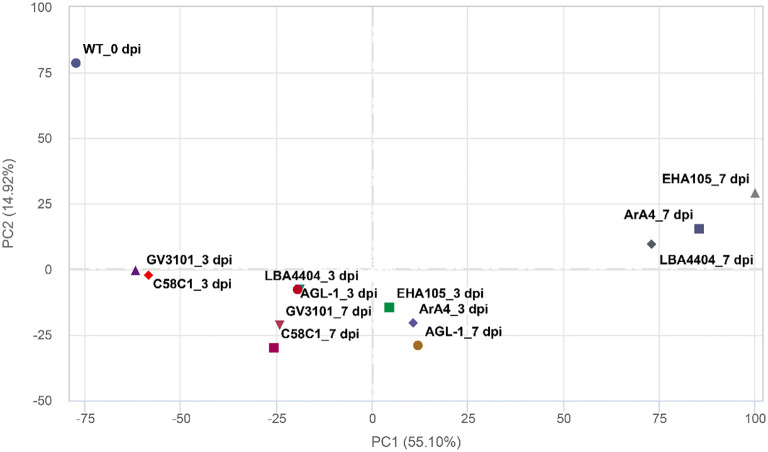
PCA revealed the transcriptome clustering of *N. benthamiana* leaves at 3 and 7 dpi after infiltration with six *Agrobacterium* strains.

**Figure 2:**
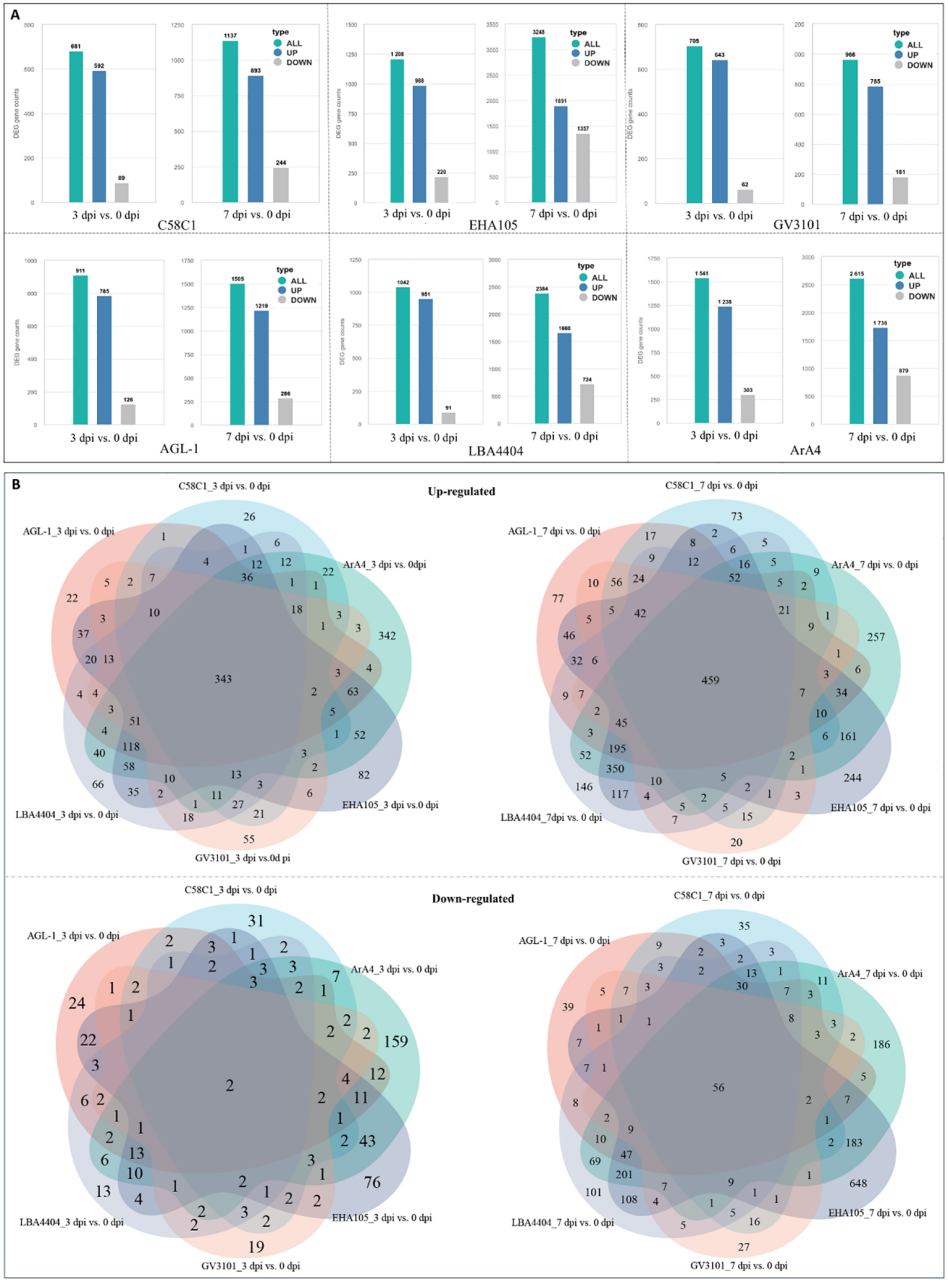
Number of DEGs, as well as shared and unique DEGs, in *N. benthamiana* leaves after infection with six different Agrobacterium strains. **(A)** The bar chart shows the total number of DEGs, as well as the numbers of upregulated and downregulated genes, in tobacco leaves at 3 dpi and 7 dpi infected with each of the six Agrobacterium strains. **(B)** Venn diagrams illustrating upregulated and downregulated DEGs in leaves infected with the six *Agrobacterium* strains at 3 dpi and 7 dpi, respectively.

To study and compare the impact of different *Agrobacterium* strains on the transcriptome of tobacco leaves during transient expression, we infiltrated tobacco leaves with 6 different *Agrobacterium* strains and conducted comparative transcriptome analysis at 3 dpi (days post-infiltration) and 7 dpi. These 6 *Agrobacterium* strains include five commonly used strains in laboratories: the *Agrobacterium tumefacien* strains C58C1, GV3101, LBA4404, AGL-1, EHA105, and the *Rhizobium rhizogene* strain ArA4. *N. benthamiana* that were not infiltrated with *Agrobacterium* were used as the control group (0 dpi). With three biological repetitions included, 39 samples were sequenced for analysis. In total, 257.02 Gb of clean bases were obtained. The number of clean bases in each sample was greater than 5.99 Gb. The percentage of Q20 bases was above 99.02%, and the percentage of Q30 bases was above 95.5%. In total, 97.29% to 99.15% of the high-quality reads were successfully mapped to the *N. benthamiana* reference genome. In total, 36,761 annotated genes were aligned in this study. Gene expression levels were normalized as fragments per kilobase of exon model per million mapped fragments (FPKM). The Spearman correlation coefficient between biological replicates for each treatment exceeded 0.80, indicating the reliability of the sampling and sequencing processes (**Supplementary Figure 1**). Principal component analysis (PCA) based on the average FPKM values revealed a distinct separation between leaves at 0 dpi and the infected leaves. Compared with those of EHA105, LBA4404 and ArA4, the transcription profiles of *N. benthamiana* infected by strains C58C1, GV3101 and AGL-1 at 3 dpi and 7 dpi showed a smaller difference (**Figure 1**). Furthermore, the expression profiles of *N. benthamiana* infected with *Agrobacterium* GV3101 and C58C1 at 3 dpi were similar. The expression profiles of *N. benthamiana* infected with *Agrobacterium* LBA4404 and AGL-1 at 3 dpi were similar. The expression profiles of *N. benthamiana* infected by *Agrobacterium* GV3101 and C58C1 at 7 dpi were similar, and they were closer to the expression profiles of *N. benthamiana* infected by LBA4404 and AGL-1 at 3 dpi. We first compared the number of differentially expressed genes (DEGs) in *N. benthamiana* induced by six *Agrobacterium* strains. At both 3 dpi and 7 dpi, all strains triggered more upregulated than downregulated DEGs, and the total number of DEGs was consistently higher at 7 dpi than at 3 dpi (**Figure 2A**). Venn diagram analysis revealed shared and unique DEGs among strains: 343 commonly upregulated and 2 commonly downregulated DEGs at 3 dpi, and 459 commonly upregulated and 56 commonly downregulated DEGs at 7 dpi. Inoculation with ArA4 led to the highest number of unique DEGs at 3 dpi, whereas EHA105 induced the most unique DEGs at 7 dpi. These results indicate distinct yet overlapping host transcriptional responses to different *Agrobacterium* strains (**Figure 2B**).

### Gene ontology and kyoto encyclopedia of genes and genomes analyses of DEGs in tobacco after infection with six different *agrobacterium* strains

**Figure 3:**
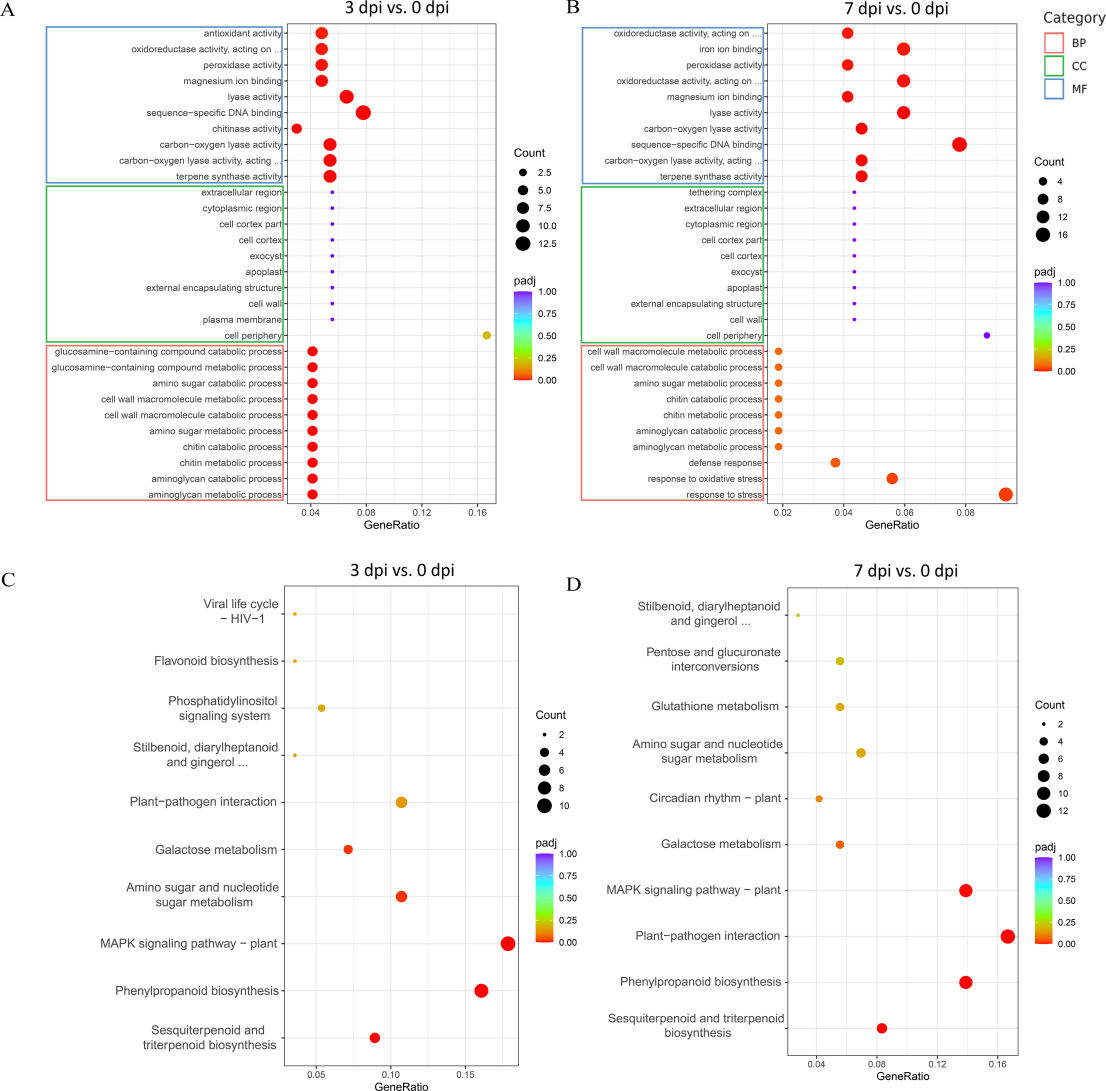
Functional and pathway enrichment analysis of common upregulated DEGs in leaves infected by six *Agrobacterium* strains at 3 dpi and 7 dpi. **(A)** GO enrichment analysis of common DEGs in leaves infected by six *Agrobacterium* strains at 3 dpi. **(B)** GO enrichment analysis of common DEGs in leaves infected by six *Agrobacterium* strains at 7 dpi. BP, Biological Process; CC, Cellular Component; MF, Molecular Function. **(C)** KEGG pathway enrichment analysis of common DEGs in leaves infected by six *Agrobacterium* strains at 3 dpi. **(D)** KEGG pathway enrichment analysis of common DEGs in leaves infected by six *Agrobacterium* strains at 7 dpi.

We conducted GO and KEGG enrichment analyses on the common DEGs that were upregulated at 3 dpi and 7 dpi in response to infection with these six different strains. The functions of genes were divided into three independent categories (molecular function, MF; biological process, BP; and cell component, CC). Bubble diagrams show the top 10 enriched GO terms in the three categories. The 343 common DEGs whose expression was upregulated at 3 dpi were significantly enriched in terpene synthase activity, carbon–oxygen lyase activity (acting on phosphates), carbon–oxygen lyase activity, chitinase activity, sequence-specific DNA binding, lyase activity, magnesium ion binding, peroxidase activity, oxidoreductase activity (acting on peroxide as an acceptor) and antioxidant activity in the MF category. The 343 DEGs were also significantly enriched in aminoglycan metabolic process, aminoglycan catabolic process, chitin metabolic process, chitin catabolic process, amino sugar metabolic process, cell wall macromolecule catabolic process, cell wall macromolecule metabolic process, amino sugar catabolic process, glucosamine-containing compound metabolic process and glucosamine-containing compound catabolic process in the BP category (**Figure 3A**). The 459 common upregulated DEGs at 7 dpi were significantly enriched in terpene synthase activity, carbon–oxygen lyase activity, phosphatase activity, sequence-specific DNA binding, carbon–oxygen lyase activity, lyase activity, magnesium ion binding, oxidoreductase activity acting on paired donors with incorporation or reduction of molecular oxygen, peroxidase activity, magnesium ion binding and oxidoreductase activity acting on peroxide as an acceptor in the MF category. The 459 DEGs were also significantly enriched in response to stress and response to oxidative stress in the BP category (**Figure 3B**). The common upregulated DEGs at 3 dpi and 7 dpi were not significantly enriched in the CC category. KEGG enrichment analysis of the 343 common upregulated DEGs at 3 dpi revealed that sesquiterpenoid and triterpenoid biosynthesis, phenylpropanoid biosynthesis, MAPK signalling, amino sugar and nucleotide sugar metabolism and galactose metabolism were significantly enriched (**Figure 3C**). Sesquiterpenoid and triterpenoid biosynthesis, phenylpropanoid biosynthesis, plant–pathogen interaction and MAPK signalling pathways were significantly enriched at 7 dpi among the 459 common upregulated DEGs (**Figure 3D**).

**Figure 4:**
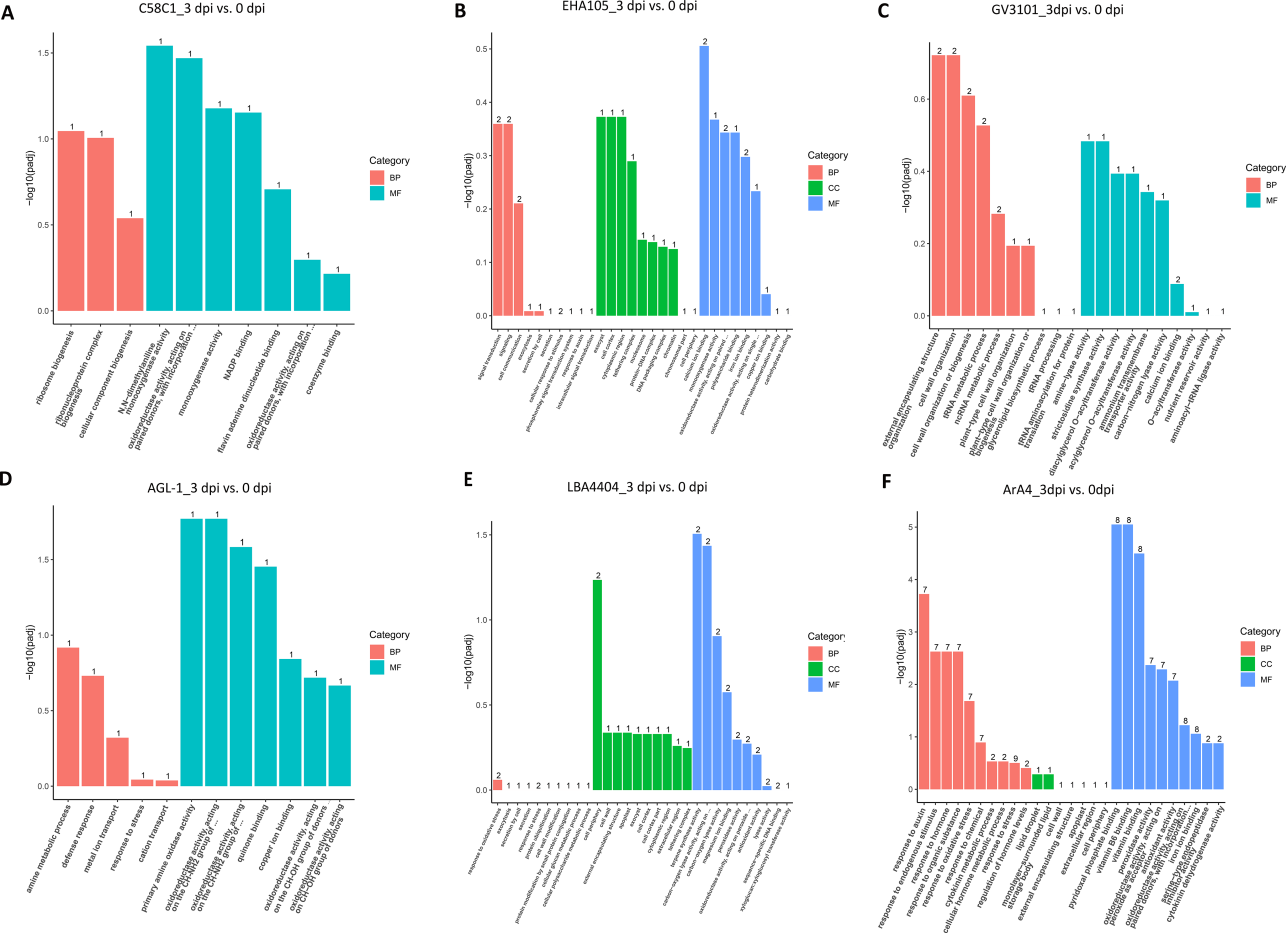
GO enrichment analysis of DEGs whose expression was specifically upregulated at 3 dpi in tobacco infected by each *Agrobacterium* strain compared with that in other strains. **(A)** GO enrichment of DEGs specifically upregulated at 3 dpi in C58C1-infected tobacco. **(B)** GO enrichment of DEGs specifically upregulated at 3 dpi in EHA105-infected tobacco. **(C)** GO enrichment of DEGs specifically upregulated at 3 dpi in GV3101-infected tobacco. **(D)** GO enrichment of DEGs specifically upregulated at 3 dpi in AGL-1-infected tobacco. **(E)** GO enrichment of DEGs specifically upregulated at 3 dpi in LBA4404-infected tobacco. **(F)** GO enrichment of DEGs specifically upregulated at 3 dpi in ArA4-infected tobacco.

We conducted GO and KEGG enrichment analyses on the genes whose expression was specifically upregulated in tobacco after infection by each strain. The diagrams show the top 10 enriched GO terms of the three categories. For C58C1 at 3 dpi, the significantly enriched GO terms were ribosome biogenesis, ribonucleoprotein complex biogenesis, N,N-dimethylaniline monooxygenase activity, oxidoreductase activity, NADP binding and flavin adenine dinucleotide binding. KEGG analysis revealed that only the tryptophan metabolism pathway was significantly enriched (**Figure 4A**; **Supplementary Figure 2A**). For EHA105 at 3 dpi, there were no significantly enriched GO terms for the specific upregulated genes. KEGG analysis revealed that protein processing in the endoplasmic reticulum (ER), endocytosis, spliceosome and long hormone signal transduction pathways were significantly enriched (**Figure 4B**; **Supplementary Figure 2B**). For GV3101 at 3 dpi, there were no significantly enriched GO terms or KEGG pathways for the specific upregulated genes (**Figure 4C**; **Supplementary Figure 2C**). For AGL-1 at 3 dpi, the significantly enriched GO terms were primary amine oxidase activity, oxidoreductase activity and quinone binding. There were no significantly enriched KEGG pathways for the specific upregulated genes (**Figure 4D**; **Supplementary Figure 2D**). For LBA4404 at 3 dpi, the significantly enriched GO terms were terpene synthase activity and carbon–oxygen lyase activity acting on phosphates. KEGG analysis revealed that sesquiterpenoid and triterpenoid biosynthesis was significantly enriched (**Figure 4E**; **Supplementary Figure 2E**). For ArA4 at 3 dpi, the significantly enriched GO terms were response to auxin, response to endogenous stimulus, response to hormone, response to organic substance, response to oxidative stress, pyridoxal phosphate binding, vitamin B6 binding, vitamin binding, peroxidase activity, oxidoreductase activity acting on peroxide as an acceptor and antioxidant activity. KEGG analysis revealed that plant hormone signal transduction, zeatin biosynthesis, cysteine and methionine metabolism, diterpenoid biosynthesis, phenylpropanoid biosynthesis, MAPK signalling, and carotenoid biosynthesis were significantly enriched (**Figure 4F**; **Supplementary Figure 2F**).

**Figure 5:**
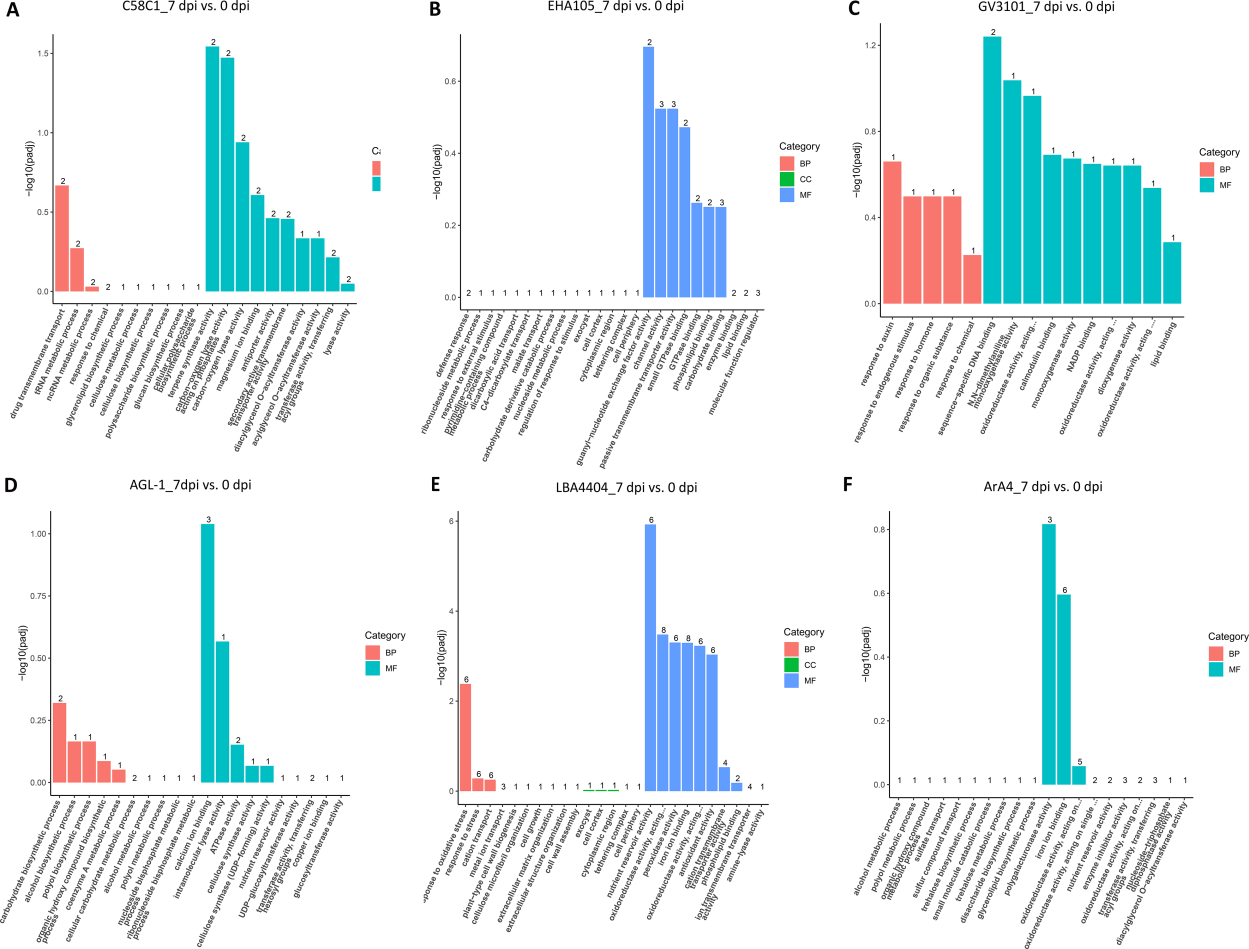
GO enrichment analysis of DEGs whose expression was specifically upregulated at 7 dpi in tobacco infected by each *Agrobacterium* strain compared with that in tobacco infected by other strains. **(A)** GO enrichment of DEGs specifically upregulated at 7 dpi in C58C1-infected tobacco. **(B)** GO enrichment of DEGs specifically upregulated at 7 dpi in EHA105-infected tobacco. **(C)** GO enrichment of DEGs specifically upregulated at 7 dpi in GV3101-infected tobacco. **(D)** GO enrichment of DEGs specifically upregulated at 7 dpi in AGL-1-infected tobacco. **(E)** GO enrichment of DEGs specifically upregulated at 7 dpi in LBA4404-infected tobacco. **(F)** GO enrichment of DEGs specifically upregulated at 7 dpi in ArA4-infected tobacco.

For C58C1 at 7 dpi, the significantly enriched GO terms were terpene synthase activity and carbon–oxygen lyase activity acting on phosphates. There were no significantly enriched KEGG pathways for the specific upregulated genes (**Figure 5A**; **Supplementary Figure 3A**). For EHA105 at 7 dpi, there were no significantly enriched GO terms or KEGG pathways for the specific upregulated genes (**Figure 5B**; **Supplementary Figure 3B**). For GV3101 at 7 dpi, there were no significantly enriched GO terms for the specific upregulated genes. KEGG analysis revealed that linoleic acid metabolism and tryptophan metabolism were significantly enriched (**Figure 5C**; **Supplementary Figure 3C**). For AGL-1 at 7 dpi, there were no significantly enriched GO terms or KEGG pathways for the specific upregulated genes (**Figure 5D**; **Supplementary Figure 3D**). With respect to LBA4404 at 7 dpi, the significantly enriched GO terms were response to oxidative stress, nutrient reservoir activity, oxidoreductase activity, peroxidase activity, iron ion binding, oxidoreductase activity acting on peroxide as an acceptor and antioxidant activity. KEGG analysis revealed that the phenylpropanoid biosynthesis pathway was significantly enriched (**Figure 5E**; **Supplementary Figure 3E**). For ArA4 at 7 dpi, no significantly enriched GO terms were detected for the specific upregulated genes. KEGG analysis revealed that the pentose and glucuronate interconversions biosynthesis pathway was significantly enriched (**Figure 5F**; **Supplementary Figure 3F**).

### Co-expression modules respond to infection by different *Agrobacterium* strains

**Figure 6:**
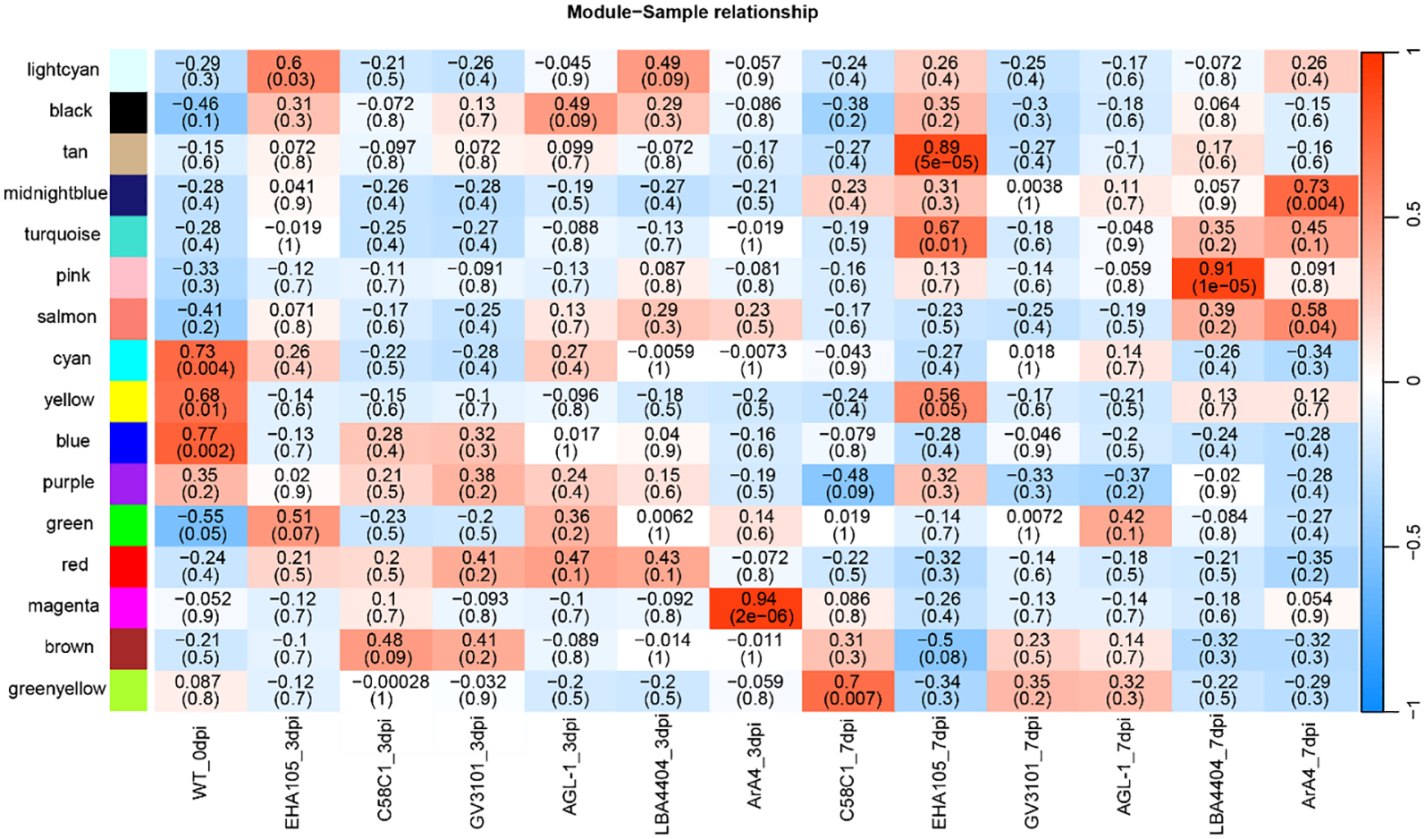
Heatmap of correlations between module eigengenes in tobacco leaves and samples infected by six distinct Agrobacterium strains at 3 dpi and 7 dpi.

To investigate gene regulatory networks underlying the *N. benthamiana* response to different *Agrobacterium* strains, weighted gene co-expression network analysis (WGCNA) was performed and 16 co-expression modules were identified. Correlation analysis showed that multiple modules were significantly associated with specific strains and time points (R² ≥ 0.5, *P* < 0.05; **Figure 6**). The yellow module was correlated with both the 0 dpi control and EHA105 at 7 dpi. Most modules showed strain- and stage-specific responses: the lightcyan (39 genes) and green (675 genes) modules were specifically associated with EHA105 at 3 dpi; the tan (149 genes) and turquoise (6901 genes) modules were strongly correlated with EHA105 at 7 dpi; the green-yellow (214 genes) module was specific to C58C1 at 7 dpi; the pink module (309 genes) was specific to LBA4404 at 7 dpi; the magenta module (289 genes) was associated with ArA4 at 3 dpi; the midnightblue (77 genes) and salmon (142 genes) modules were specific to ArA4 at 7 dpi; and the cyan (83 genes) and blue (5011 genes) modules were correlated with the 0 dpi control. Gene expression patterns of these modules are shown in **Supplementary Figure 4**.

We used the cytoHubba tool to screen the top 10 hub genes in each module regulatory network based on the maximum clique centrality (MCC) method (**Supplementary Figure 5**). The top 10 hub genes from the cyan, yellow, and blue modules associated with the 0-dpi group participate in diverse biological processes. These processes include abiotic stress responses, redox homeostasis, primary metabolism, cell wall and cuticle formation, chromatin remodelling, transcriptional and post-transcriptional regulation, cytoskeleton dynamics, and plant growth and development. These three modules modulate plant stress resistance, metabolic homeostasis, and growth processes. They therefore contribute substantially to plant environmental adaptation, cellular stability maintenance, and developmental regulation. The hub genes of the lightcyan module are specifically correlated with the EHA105_3dpi group. These genes are primarily involved in lipid transport, cuticle and wax biosynthesis, fatty acid elongation, cell wall modification, and stress responses. These hub genes reshape the plant epidermal cell wall and cuticle barrier. The dynamic modification of cell wall structure reduces physical defense barriers, facilitates *Agrobacterium* attachment and invasion, and promotes the initial delivery of T-DNA complexes into plant cells, thereby positively regulating the early progress of T-DNA transfer. The magenta module is specifically associated with the ArA4_3dpi group. Its core hub genes mainly participate in auxin signal transduction, cell elongation, plant growth and development, as well as stress and defense responses. Auxin signaling activation mediated by these hub genes modulates intracellular physiological status and alleviates excessive immune stress triggered by early *Agrobacterium* infection, which stabilizes the intracellular microenvironment and prevents premature cell defense responses from inhibiting T-DNA nuclear import. The hub genes in the green–yellow module correspond to the C58C1_7dpi group. They are mainly responsible for purine transport, calcium signalling, lipid metabolism, RNA post-transcriptional regulation, and abiotic stress responses. At the late infection stage of C58C1, these hub genes regulate calcium signal transduction and RNA post-transcriptional modification. Calcium signaling fine-tunes plant immune homeostasis to avoid excessive stress response blocking exogenous gene expression, while RNA regulatory genes optimize the transcription efficiency of T-DNA-derived exogenous genes, indirectly promoting the accumulation of target proteins during transient transformation. The tan module hub genes are specifically correlated with EHA105_7dpi. They function in vacuolar protein processing, auxin signalling, chloroplast stress response, redox homeostasis, the MAPK cascade, and protein ubiquitination. These late-stage hub genes primarily govern protein turnover and stress signal balancing during transient transformation. The protein ubiquitination and vacuolar processing pathways precisely regulate the stability and degradation of exogenous proteins, preventing excessive protein aggregation and dysfunction; meanwhile, MAPK cascade and redox homeostasis regulation relieve infection-induced oxidative damage, ensuring sustained and efficient accumulation of functional exogenous proteins. The turquoise module hub genes are associated with the EHA105_7dpi group. They primarily regulate chloroplast homeostasis, ethylene signal transduction, protein degradation, vacuolar transport, kinase-mediated signalling, and nucleic acid metabolism. Notably, these core hub genes modulate nucleic acid metabolism and intracellular signal transduction to further optimize host chromatin accessibility at the late transformation stage. The dynamic balance of protein degradation and transport eliminates misfolded proteins generated during high-level exogenous expression, maintaining cellular homeostasis and guaranteeing the continuous and stable expression and accumulation of T-DNA-carried exogenous genes. The pink module hub genes correspond to the LBA4404_7dpi group. They are involved in secondary metabolism, zinc and copper ion transport, signal transduction, and transcriptional regulation. These core genes control metal homeostasis and enhance plant environmental adaptation capacity. The midnightblue module hub genes are specifically correlated with ArA4_7dpi. They mainly mediate heat shock responses, protein folding and stabilization, and calcium-dependent stress signalling. As key regulators of protein homeostasis during late ArA4 infection, these hub genes encode molecular chaperones that assist correct folding of exogenous proteins synthesized in transient transformation, reduce protein denaturation and degradation under infection stress, and effectively improve the accumulation level of functional exogenous proteins. The salmon module hub genes are also associated with the ArA4_7dpi group. They participate in RNA processing, vesicular transport, chromosome stability, intracellular trafficking, and redox regulation. These hub genes facilitate efficient RNA processing of T-DNA-derived transcripts to ensure normal transcription of exogenous genes. Moreover, vesicular and intracellular trafficking systems mediate the transport and localization of newly synthesized exogenous proteins, while chromosome stability maintenance sustains persistent transcriptional activity of transformed genes, collectively supporting continuous and efficient transient protein expression.

### Identification of genes affecting the expression levels of recombinant proteins

**Figure 7:**
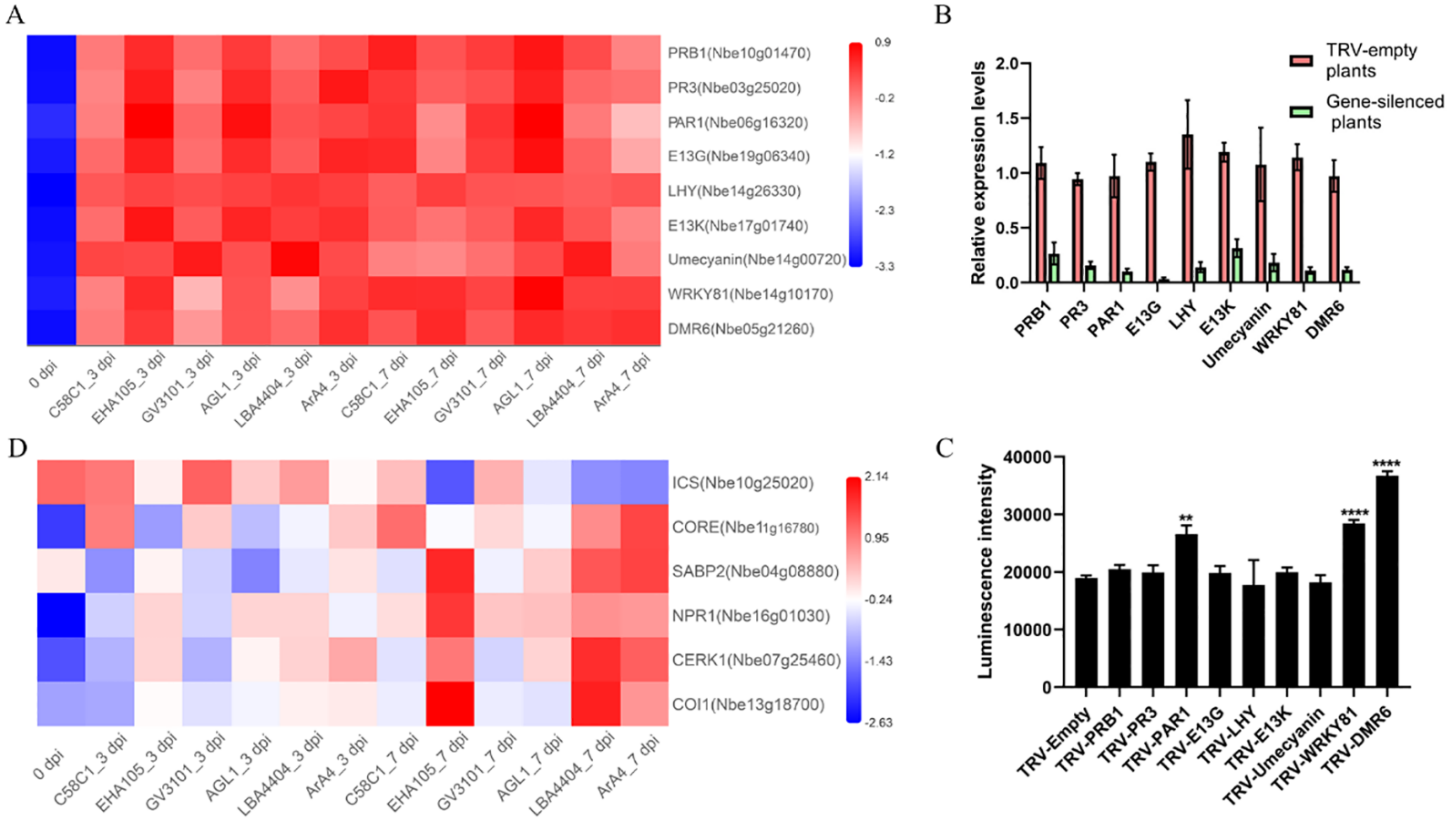
Identification of genes associated with recombinant protein expression levels. **(A)** Heatmap of expression levels for genes significantly upregulated in tobacco leaf transcriptomes after infection by all six *Agrobacterium* strains. **(B)** Detection of virus-induced gene silencing efficiency by qPCR. **(C)** Comparison of luminescence intensity between target gene-silenced plants and empty vector control plants after luciferase transient expression mediated by C58C1 *Agrobacterium.* **(D)** Expression levels of previously reported immune-related genes that regulate recombinant protein expression in tobacco leaf transcriptomes after infection by six *Agrobacterium* strains. **p ≤ 0.01; ****p ≤ 0.0001.

After infection with *Agrobacterium*, the expression of defence-related genes is activated in tobacco leaves. The defence mechanism may be among the important factors that hinder exogenous protein expression through multiple pathways, thus inhibiting the accumulation of recombinant proteins. With respect to the transcriptome data, we screened defence-related genes whose expression levels were rapidly and significantly upregulated at 3 dpi after infection with each *Agrobacterium* strain (**Figure 7A**) and explored the effects of these genes on exogenous protein expression. These 9 genes are pathogenesis-related protein 1b (*PRB1*), glycosyl hydrolase superfamily protein (*PR3, E13K* and *E13G*), phytochrome rapidly regulated 1 (*PAR1*), late elongated hypocotyl (*LHY*), umecyanin, *WRKY81* and *DMR6*. These nine genes and the *PDS* gene were silenced in tobacco using virus-induced gene silencing (VIGS). When the *PDS*-silenced tobacco plants displayed a bleached phenotype (**Supplementary Figure 6**), successful silencing of all nine target genes was confirmed by qPCR (**Figure 7B**). At this stage, the LUC reporter gene was transiently expressed in the gene-silenced plants. Compared with that in the TRV−empty control plants, the fluorescence intensity in the *PAR1*-, *WRKY81*-, and *DMR6*-silenced plants was significantly greater (**Figure 7C**). Furthermore, we analysed the expression profiles of six previously reported genes that negatively regulate heterologous protein accumulation from the transcriptome data (**Figure 7D**). The expression levels of these genes were subsequently validated by qPCR (**Supplementary Figure 7**), and the results were consistent with the transcriptome data, confirming the reliability of our transcriptome analysis. These six genes exhibited distinct response patterns to the six different *Agrobacterium* strains. The expression of *ICS* was highest and comparable at 0 dpi and in the GV3101_3 dpi samples but decreased in all the other samples. The transcript levels of *CORE*, *NPR1*, *CERK1*, and *COI1* were upregulated at 3 and 7 dpi across all *Agrobacterium* infiltrations. The expressions of *NPR1* and *CERK1* were greater at 7 dpi than at 3 dpi for all the strains. *SABP2* expression was downregulated at 3 dpi but induced at 7 dpi following infiltration with all the strains except C58C1. Overall, compared with those of the other *Agrobacterium* strains, the expression of most genes was strongly induced in response to EHA105, LBA4404, and ArA4.

## Discussion

Both the strain genetic background and modified Ti plasmid combinations significantly shape the host compatibility and transformation performance of disarmed *Agrobacterium* in various plant materials (Hwang et al., 2010, 2013). Even Genetic variations among isogenic *Agrobacterium* strain derivatives are well documented to alter host transformation efficiency (De Saeger et al., 2021), yet the underlying transcriptomic discrepancies induced by commonly used laboratory strains remain poorly characterized. Understanding the transcriptomic responses of *N. benthamiana* to different. The selection of appropriate *Agrobacterium* strains according to the properties of various recombinant proteins plays a vital role in improving the efficiency of transient transformation for biotechnological applications. The present study systematically characterized the dynamic transcriptomic responses of *N. benthamiana* to six widely used *Agrobacterium* strains, clarifying how they reshape host transcriptional reprogramming. These results fill a key knowledge gap regarding the variable host responses triggered by different *Agrobacterium* strains and provide targeted theoretical guidance for strain selection and transient transformation optimization in plant biotechnology.

PCA revealed that the transcriptomic profiles of *N. benthamiana* at 3 and 7 dpi differed only slightly following infection with strains C58C1, GV3101 and AGL-1. In contrast, obvious temporal divergence was observed between 3 and 7 dpi in leaves infiltrated with strains EHA105, ArA4 and LBA4404. These transcriptomic results demonstrate for the first time that commonly used *Agrobacterium* strains fall into two distinct host interaction patterns: C58C1, GV3101 and AGL-1 induce rapid host transcriptional homeostasis with stable immune and metabolic states at the late infection stage, while EHA105, ArA4 and LBA4404 trigger persistent and dynamic transcriptomic reprogramming throughout infection. In terms of strain background, the *Agrobacterium* strains C58C1, GV3101, AGL-1, and EHA105 share the same C58 genetic origin. Although all of them are derived from *Agrobacterium* C58, certain genetic differences still exist among these isogenic strain backgrounds (De Saeger et al., 2021). Notably, strains AGL-1 and EHA105 harbor identical helper plasmids; however, they exhibit significant differences in the temporal patterns of tobacco transcriptomes. This confirms that chromosomal genetic variations independently regulate host response dynamics, independent of Ti plasmid background. For instance, strain AGL-1 contains an intact recA gene in its chromosomal background, whereas strain EHA105 is a recA-deficient strain (Lazo et al., 1991; Hood et al., 1993). Strains GV3101 and EHA105 differ in their helper Ti plasmids, which may also further diversify *Agrobacterium*-host interaction dynamics. In summary, distinct *Agrobacterium* strains establish different interaction patterns with the host during infection: some strains can quickly reach a balance with the host, whereas others drive the host to undergo continuous transcriptional reprogramming. A recent large-scale agrobacterial screening study systematically evaluated the transient expression capacity of multiple *Agrobacterium* strains in *N. benthamiana*. Strains including C58C1, GV3101, AGL-1, EHA105 and LBA4404 exhibited comparable levels of transient expression, while the ArA4 strain showed superior performance (LopezAgudelo et al., 2025). Nevertheless, molecular differences induced by these strains in host plants have not been systematically characterized. The transcriptomic differences identified in our study reveal long-overlooked molecular differences among *Agrobacterium* strains in their interactions with host plants.

Combining the results of GO functional enrichment and KEGG pathway analyses of common DEGs, the present study identified a conserved two-stage temporal response pattern in *N. benthamiana* upon infection with diverse *Agrobacterium* strains. At the early infection stage (3 dpi), host DEGs were predominantly enriched in pathways related to cell wall reconstruction, aminoglycan and carbohydrate metabolism, and antioxidant defense, accompanied by the activation of MAPK signaling and secondary metabolite biosynthesis. These results indicate that *N. benthamiana* employs an early adaptive molecular strategy centered on physical barrier reinforcement and metabolic reprogramming to cope with *Agrobacterium* colonization. At the late infection stage (7 dpi), the host underwent a distinct functional transition: metabolic and cell wall-related responses were largely diminished, while plant–pathogen interaction and oxidative stress defense pathways were strongly activated. This shift reflects a second universal molecular strategy, in which the host prioritizes intensive immune activation to resist persistent bacterial infection. This conserved temporal regulatory cascade is intrinsic to tobacco responses to *Agrobacterium* and is largely independent of different strains. Functionally, the metabolically active state at 3 dpi favors transient exogenous gene expression, whereas the elevated immune response at 7 dpi molecularly explains the commonly observed decline in recombinant protein accumulation at the late infiltration stage. Notably, beyond these universal host responses, strain-specific DEG enrichment further revealed divergent, strain-dependent molecular strategies that differentiate host transcriptional reprogramming triggered by distinct *Agrobacterium* strains. C58C1 induced a temporal regulatory shift from early oxidative metabolism to late terpenoid biosynthesis, representing a unique metabolic transition strategy. AGL-1 elicited only mild and early-stage responses related to amine oxidation and quinone binding, with no obvious functional remodeling at the late stage. LBA4404 drove a clear functional switch from early terpenoid synthesis to late oxidative stress response. Differently, ArA4 specifically and broadly activated hormone signaling and oxidative stress pathways exclusively at the early infection stage. In contrast, GV3101 and EHA105 induced relatively stable and non-differentiated host functional responses throughout infection, with negligible temporal variation. These findings provide crucial insights into the molecular strategies underlying the differential host–*Agrobacterium* interactions.

In addition to the transcriptional transition from metabolic adaptation to immune activation, the progressive decline of recombinant protein accumulation at the late stage of *Agrobacterium*-mediated transient transformation is largely attributed to the crosstalk between oxidative stress, ER stress, and disrupted cellular proteostasis. *Agrobacterium* infiltration triggers host immune activation and sustained reactive oxygen species (ROS) burst, which disrupts intracellular redox balance and induces oxidative damage to newly synthesized proteins. During oxidative protein folding, the ER also generates ROS (Ali et al., 2025). Meanwhile, the massive and rapid synthesis of heterologous recombinant proteins substantially overloads the folding and processing capacity of the ER lumen, inevitably inducing severe ER stress and activating the unfolded protein response (UPR) pathway (Hamel et al., 2024). Persistent ER stress further exacerbates ROS accumulation, forming a vicious cycle that severely impairs cellular protein homeostasis (Cao et al., 2022). Under prolonged stress conditions, misfolded and damaged recombinant proteins cannot be efficiently repaired by ER-resident chaperones such as BiP and calnexin, and are ultimately eliminated through enhanced ER-associated degradation and vacuolar proteolysis pathways. Göritzer et al. demonstrated that ER expansion via CCT gene editing in *N. benthamiana* promoted BiP accumulation and recombinant protein production, significantly increasing cellular tolerance to ER stress (Göritzer et al., 2025).

Plant innate immune responses strongly restrict *Agrobacterium*-mediated transient transformation. Studies in Ageratum conyzoides revealed that stronger plant defence responses are correlated with lower *Agrobacterium* transformation efficiency, while *Agrobacterium* can suppress host immunity to facilitate infection (Ditt et al., 2005). Plants recognize conserved microbial components via pattern recognition receptors such as the LRR receptor kinase EFR, which detects the elongation factor EF−Tu of *Agrobacterium tumefaciens* and activates downstream defence signalling. Arabidopsis efr mutants exhibit impaired immune activation towards *Agrobacterium* and display enhanced susceptibility to transient transformation (Zipfel et al., 2006). Suppression of plant immunity further improves transformation efficiency: the bacterial effector AvrPto inhibits multiple pattern recognition receptors to attenuate host defence; when conditionally expressed in Arabidopsis via a dexamethasone-inducible system, AvrPto markedly increases transient expression after agroinfiltration (Tsuda et al., 2012). In addition, salicylic acid (SA)-dependent defence signalling constrains transient transformation. Reducing endogenous SA accumulation by expressing the SA-degrading enzyme NahG also significantly increases the *Agrobacterium*-mediated transient transformation efficiency. Collectively, suppression or impairment of host immune pathways effectively relieves defence-related inhibition and thus facilitates transient expression induced by *Agrobacterium* (Rosas-Díaz et al., 2017). In this study, three defence-related genes that regulate the expression level of recombinant proteins were identified. *PAR1* acts as a core hub that integrates light signals with the jasmonic acid (JA) defence hormone signalling pathway and functions as a coordinator that mediates resource allocation between plant growth and defence (Liu et al., 2019). Umecyanin belongs to the stellacyanin subclass of plant phytocyanins and plays an essential regulatory role in plant resistance against phytopathogens (Zhu et al., 2018; Liu et al., 2020). *WRKY* genes are widely involved in the response to biotic stress and perform positive regulatory functions during defence against pathogen infection (Wang et al., 2026). DMR6 (*DOWNY MILDEW RESISTANT 6*) acts as a key negative regulator of plant immunity. In *Arabidopsis* and other plant species, it encodes a 2-oxoglutarate/Fe(II)-dependent dioxygenase, which fine-tunes the balance between disease resistance and growth by modulating the salicylic acid (SA) metabolic pathway (Zhang et al., 2017). Silencing these genes to suppress plant immunity enhances recombinant protein production. This is a simple and universal optimization strategy for the use of tobacco as a bioreactor.

All transcriptomic profiles in this study were obtained from bulk leaf tissues, which only reflect averaged gene expression signals and cannot resolve potential cell-type-specific transcriptional heterogeneity during *Agrobacterium* infection. Accumulating evidence indicates that plant immune and stress responses to pathogen invasion are spatially compartmentalized and cell-specific, whereby epidermal, mesophyll, and vascular cells exhibit distinct stress perception, signal transduction, and defence reprogramming behaviours, and localized defensive microdomains at infection sites are often masked in bulk RNA-seq analyses (Ali et al., 2026). Therefore, the strain-specific immune and metabolic reprogramming observed in our present study may conceal divergent responses among different cell populations. Future studies combining single-cell/nucleus RNA sequencing and spatial transcriptomics will help precisely resolve cell-type-specific regulatory events during plant–*Agrobacterium* interaction, uncover the fine-scale spatiotemporal patterns of T-DNA transformation and host defence activation, and further deepen our mechanistic understanding of strain-dependent transformation efficiency differences at high cellular resolution.

In summary, this study performed a transcriptomic comparison of tobacco infected with six different *Agrobacterium* strains and systematically revealed the divergent characteristics of host transcriptional reprogramming induced by genetic differences among strains. We clarified the differential regulation of immune pathways and stress responses in tobacco by distinct *Agrobacterium* strains and elucidated the strain-specific effects on host transcriptional profiles at the molecular level. This work highlights the similarities and differences in host transcription following infection by commonly used laboratory *Agrobacterium* strains. Taken together, these findings provide comprehensive transcriptomic evidence and a fundamental theoretical basis for selecting optimal engineered strains and precisely modifying host defence pathways to improve recombinant protein production in plant bioreactors. Our findings fill the knowledge gap regarding the strain-specific temporal regulatory mechanisms of *Agrobacterium*–plant interactions, and provide novel mechanistic insights and theoretical references for rational strain selection and optimization of transient transformation systems.

## Materials and methods

### Plant materials and *Agrobacterium* infiltration of *N. benthamiana*

Wild-type *N. benthamiana* plants were grown in a greenhouse. The growth temperature was 25 °C, the humidity was controlled at 40–50%, the light intensity was 200 μmol/m²·s, and the light cycle was 16 LH/8 DH. Five-week-old plants were used for *Agrobacterium* infiltration. Six different *Agrobacterium* strains were cultured overnight at 28 °C/250 rpm in LB medium (10 g/L tryptone, 5 g/L yeast extract, and 10 g/L NaCl; pH = 7.0) supplemented with the corresponding antibiotics. The medium for culturing C58C1 contained 50 μg/L rifampicin, 50 μg/L carbenicillin and 50 μg/L tetracycline. The medium for culturing EHA105 contained 50 μg/L rifampicin. The medium for culturing GV3101 contained 50 μg/L rifampicin and 50 μg/L gentamicin. The medium for culturing LBA4404 contained 50 μg/L rifampicin and 50 μg/L streptomycin. The medium for culturing AGL-1 contained 50 μg/L rifampicin and 50 μg/L carbenicillin. The medium for culturing ArA4 contained 50 μg/L kanamycin. The bacteria were resuspended in infiltration medium (10 mM MES, 10 mM MgCl_2_, 150 μM acetosyringone, pH 5.6) to an OD (600 nm) of 1. The plants were then agroinfiltrated with *Agrobacterium* suspension using a needleless syringe and grown in a greenhouse at 200 μmol/m²·s light and 21 °C and 40–50% relative humidity.

### RNA-seq and read processing

3 dpi and 7 dpi were selected for transcriptome sequencing as they represent two critical stages during *Agrobacterium*-mediated transient expression. At 3 dpi, the host transcriptome is fully engaged in the initial immune response triggered by *Agrobacterium* perception and T-DNA delivery. This time point is widely used for assessing early defence activation. At 7 dpi, recombinant protein accumulation typically peaks, and host transcriptional reprogramming becomes fully established (Grosse-Holz et al., 2018). Agroinfiltrated leaves at 3 dpi and 7 dpi were sampled, immediately frozen in liquid nitrogen, and stored at -80 °C in an ultra-low temperature freezer. Non-agroinfiltrated leaves were sampled at 0 dpi as the control group. Total RNA was extracted, and genomic DNA was removed using an RNA Easy Fast Kit (DP452; TIANGEN Co. Ltd., Beijing, China) following the manufacturer’s instructions. After quality control, RNA-seq libraries were constructed using the NEBNext^®^ Ultra™ II RNA Library Prep Kit for Illumina^®^ (NEB, Ipswich, MA, USA). Libraries were sequenced with 150-bp paired-end reads on the Novaseq 6000 system according to the manufacturer’s protocol to generate transcriptome data. Adapter sequences and low-quality reads were removed using FastQC software (v0.12.1) to obtain clean reads. Clean reads were aligned to the lifenglab_nicotiana_benthamiana_v1_0 reference genome using Hisat2 software (v2.0.5). Gene expression levels were quantified using featureCounts (v1.5.0-p3). On the basis of the read counts obtained for each gene, fragments per kilobase of transcript per million mapped reads (FPKM) values were calculated to normalize gene expression levels for subsequent comparative and functional analyses. Novel gene prediction was performed using StringTie (v1.3.3b). StringTie uses a network flow algorithm and optional *de novo* assembly to assemble transcripts.

### Transcriptome analysis

DEGs between agroinfiltrated leaves and noninfiltrated samples were identified using DESeq2 software (v1.20.0). The genes whose |log2fold change (log2FC)| was ≥6 and padj (adjusted p value) was ≤0.05 were identified as DEGs. GO and KEGG enrichment analyses were conducted using clusterProfiler (v3.8.1). GO terms or KEGG pathways whose padj was ≤0.05 (or -log_10_(padj)≥1.301) were considered significantly enriched. The WGCNA (v1.7.0) package in R (v4.3.0) was employed to conduct weighted correlation network analysis with a soft-thresholding power (β) of 20 and a coefficient R² for scale-free networks = 0.8. Co-expression modules were identified using the automatic network construction function. The clustering tree was cut into distinct modules using the dynamicTreeCut method, with a minimum module size of 30 genes. Modules with a correlation coefficient of > 0.75 were merged. Co-expression networks were visualized using Cytoscape software (version 3.10.4).

### Virus-induced Gene Silencing

The 300 bp specific fragment of the CDS was cloned using specific primers and then ligated into the TRV2 vector (predigested with BamHI and XmaI) via homologous recombination. The sequences of the primers used to clone the target fragments of each gene are shown in **Supplementary Table 1**. Equivalent volumes of *Agrobacterium* C58C1 strains harbouring TRV1 and TRV2 vectors (adjusted to OD_600_ = 1) were mixed and infiltrated into the leaves of two-week-old *N. benthamiana* following the method described above. Two weeks later, when the leaves of the TRV2-P*DS* plants exhibited bleaching, the silencing efficiency of the other target genes was determined by qPCR.

### Fluorescence intensity measurement

In *N. benthamiana* plants with successful target gene silencing, leaves were infiltrated with *Agrobacterium* C58C1 harbouring the 35S::LUC construct. At 3 dpi, 6−mm leaf discs were punched from the infiltrated leaves. Luciferase protein was extracted using an Enhanced Firefly Luciferase Reporter Gene Assay Kit (Beyotime, Shanghai, China), added to white opaque 96-well plates, and incubated with firefly luciferin substrate to trigger luminescence. The fluorescence intensity was subsequently detected at 560 nm using a SpectraMax i3x multimode microplate reader. Three biological replicates were performed.

### Gene expression analysis

RNA from *N. benthamiana* leaves was isolated using an RNA Extraction Kit (TIANGEN, Beijing, China) according to the manufacturer’s instructions. The extracted total RNA was reverse transcribed into cDNA using the TIANScript II cDNA First Strand Synthesis Kit (KR107; TIANGEN, Beijing, China) according to the manufacturer’s instructions. Gene expression levels were analysed by qPCR on an Applied Biosystems QuantStudio1 (Thermo Fisher Scientific, Waltham, MA, USA) using 2 × ChamQ Blue Universal SYBR qPCR Master Mix (Vazyme, Nanjing, China). The housekeeping gene *Actin* was used as the reference gene. The primers used for the qPCR of each gene are shown in **Supplementary Table 1**.

## Data Availability

The data presented in the study are deposited in the ScienceDB repository which can be accessed at https://doi.org/10.57760/sciencedb.40003.
