# Supplementary material for: Transcriptomic analysis revealed distinct transcriptional responses in N. benthamiana upon infiltration with different Agrobacterium strains
Source: Front Plant Sci. 2026 Jul 8;17:1868449. doi: 10.3389/fpls.2026.1868449 (PMC13388453; doi:10.3389/fpls.2026.1868449)
Supplement: Supplementary file 1 [file Table1.docx]

**Table S1 Primer sequences**

| Use | Primer | Forward primer sequence | Reverse primer sequence |
| --- | --- | --- | --- |
| Clone | TRV-PRB1 | agaaggcctccatggggatccATGGGATTTGTTCTCTTTTC | tgtcttcgggacatgcccgggGGCCTTAGCAGCCGTCATGA |
| Clone | TRV-PR3 | agaaggcctccatggggatccTGGTTCCCTGAGTGCAGAAC | tgtcttcgggacatgcccgggCGATTCGCCGCCTGATCCGC |
| Clone | TRV-PAR1 | agaaggcctccatggggatccATGGCTTCATTCCACAGCTT | tgtcttcgggacatgcccgggATCAGAAGAAATGCCGAGGG |
| Clone | TRV-E13G | agaaggcctccatggggatccATTGCCAACAATTTACCATC | tgtcttcgggacatgcccgggAGCAACAAATGGTGCATATT |
| Clone | TRV-LHY | agaaggcctccatggggatccTTAGAAAAGGAGGCTGTTAT | tgtcttcgggacatgcccgggTAACCCTTGGGAGCAATTTT |
| Clone | TRV-E13K | agaaggcctccatggggatccTGGTGTCCCAAATCAAGATA | tgtcttcgggacatgcccgggAATTCTTCGCGAAAAATACT |
| Clone | TRV- Umecyanin | agaaggcctccatggggatccGGCCAGAAATTAGCTATTACA | tgtcttcgggacatgcccgggTTAAAGAAAAACGGCCAAGAC |
| Clone | TRV- WRKY81 | agaaggcctccatggggatccATCGACGGGTTAATTCGTGG | tgtcttcgggacatgcccgggGCCATCATCCACCAAATCAG |
| Clone | TRV-DMR6 | agaaggcctccatggggatccATGGAAACAAAAGTTCTGTCC | tgtcttcgggacatgcccgggATCTGAGTACAATTTCAACTT |
| qPCR | qPRB1 | ACAGCTCGTGCAGATGTAGG | GCAGCCGTCATGAAATCGTC |
| qPCR | qPR3 | TCGGCAAAATGACCAGAGTGA | ATGGCTTGTTGTCCTGTGCT |
| qPCR | qPAR1 | CCACAGCTTAAAGACATTGGC | CTTCTTCCCCACTCCTTCGC |
| qPCR | qE13G | TGCTACTTACTGAAGCACCCTT | CCCATCCATTGGCTCTTGAAAC |
| qPCR | qLHY | GTCCCAATAAGTCAAGCACTCA | TCAGCAATCGGTTCTTTCTCCA |
| qPCR | qE13K | GGGAAGGAAGAGGGCACAAT | TTGACGAATTCGCTAGGGCT |
| qPCR | qUmecyanin | GCATTGCCAAAATGGCCAGAA | GATTAGCACCACCACCAAAAGT |
| qPCR | qWRKY81 | TGGATACTCCTATTCATGGCGATT | TGATCATCGTTGGCTAGTAGGG |
| qPCR | qDMR6 | GTTCTGTCCGGAATTCGCCA | CCGAGCTGTCTCTCGCATTT |


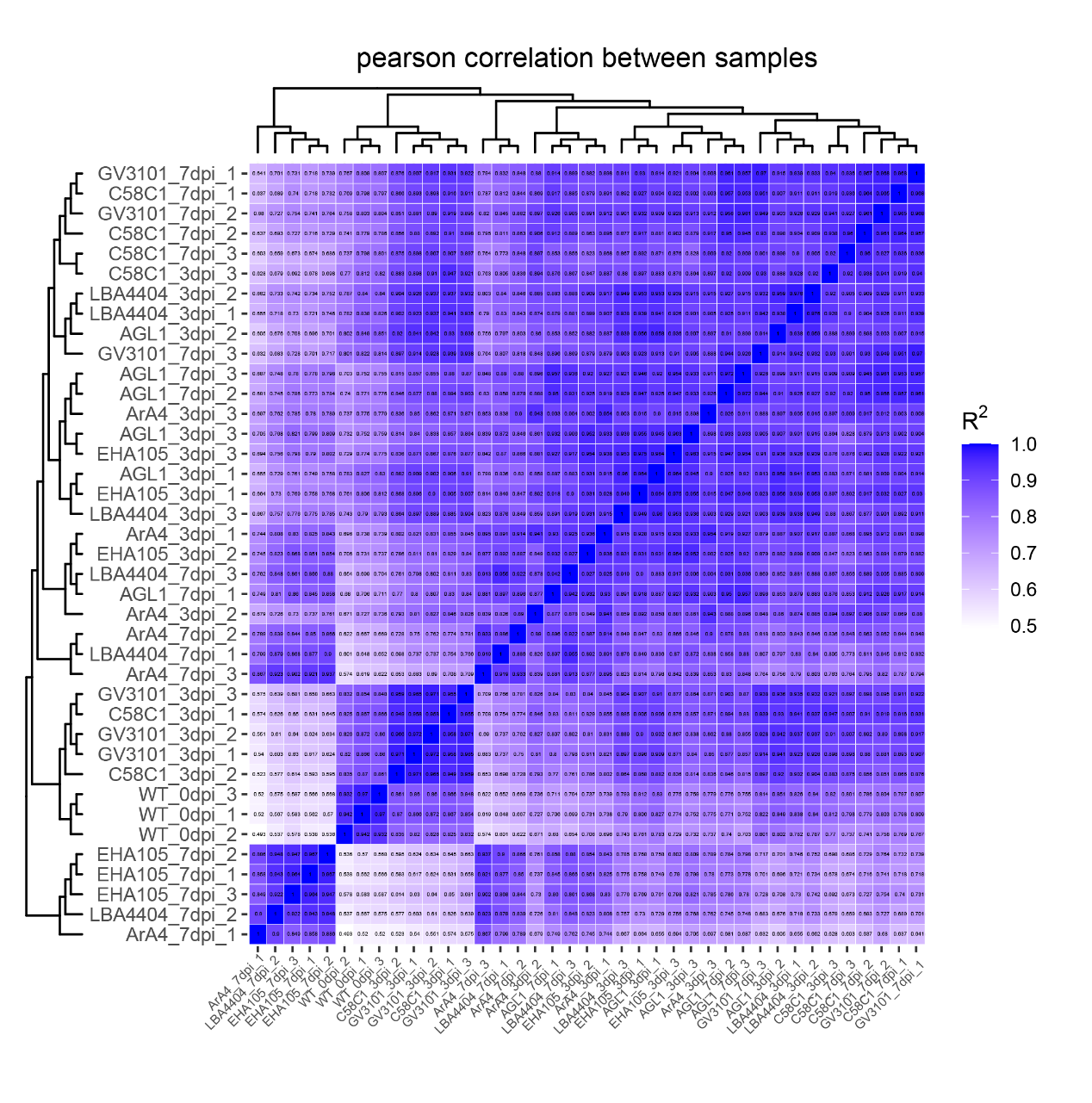


**Figure S1** Correlation analysis between all transcriptome samples. 1, 2, and 3 represent three biological replicates. Correlation coefficients are shown in boxes, with color scales representing the correlation coefficients.


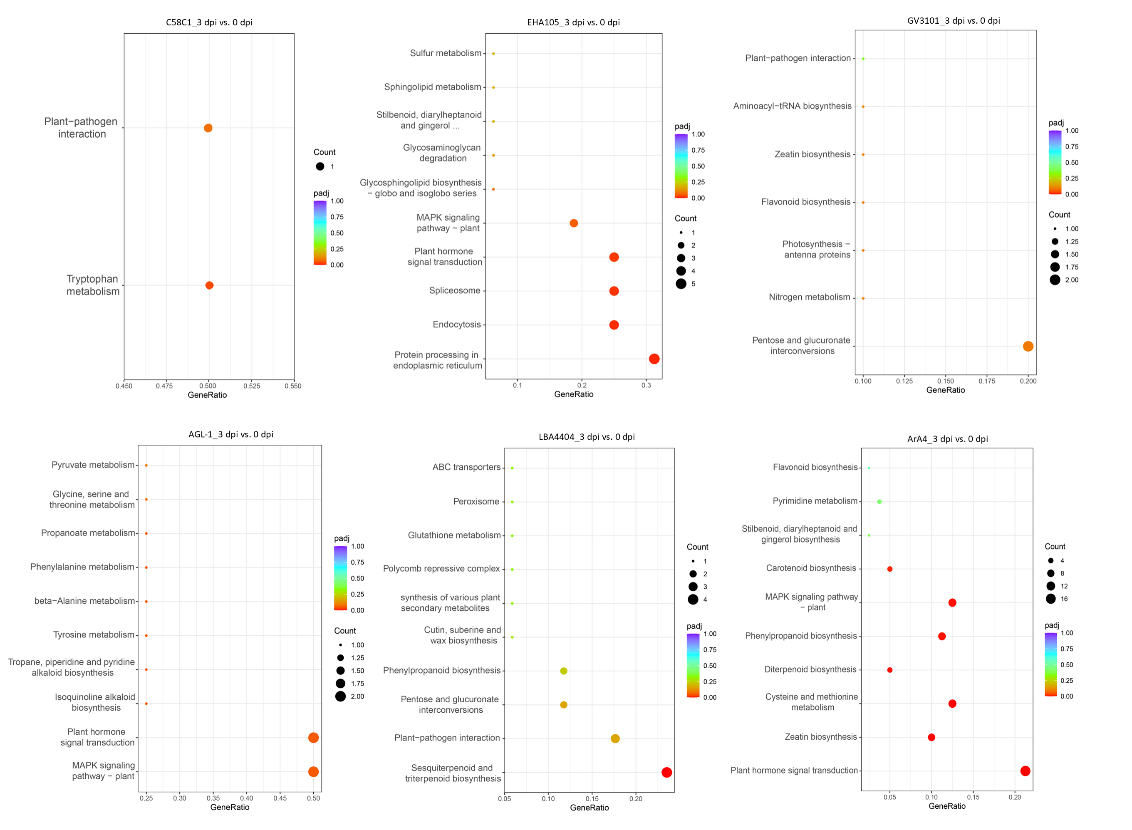


**Figure S2** KEGG pathway enrichment analysis of specifically upregulated DEGs at 3 dpi in tobacco infected by each *Agrobacterium* strain compared with other strains.


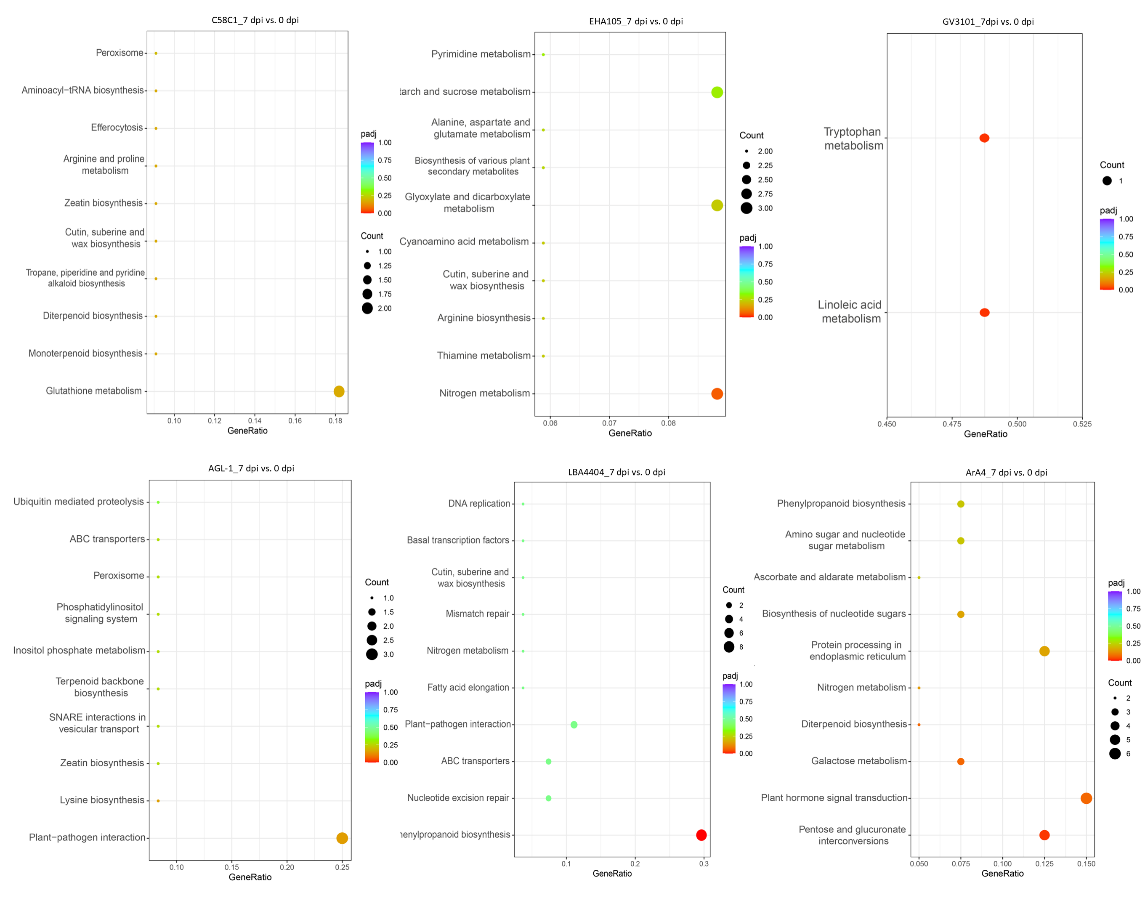


**Figure S3** KEGG pathway enrichment analysis of specifically upregulated DEGs at 3 dpi in tobacco infected by each *Agrobacterium* strain compared with other strains.


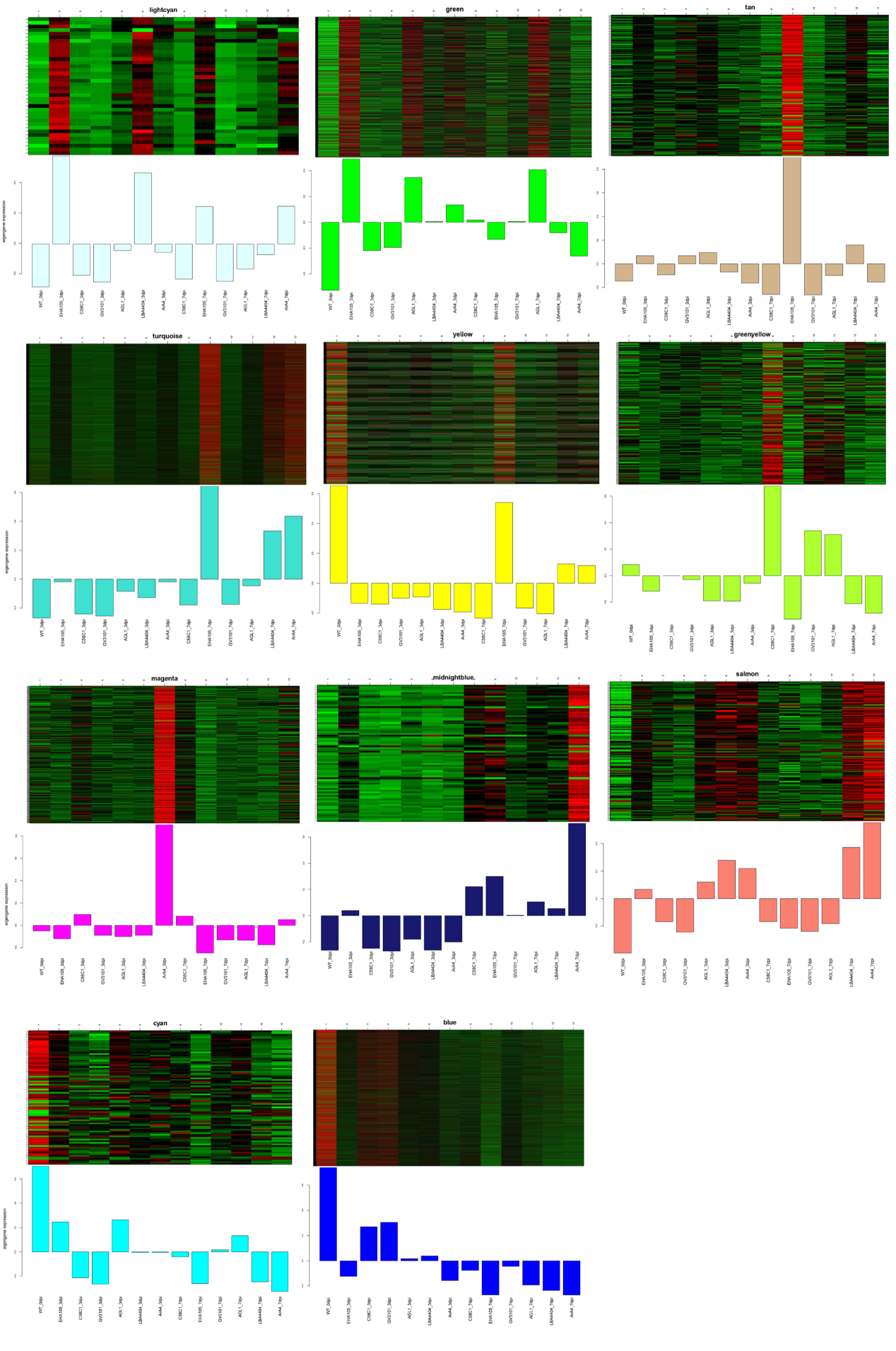


**Figure S4** Gene expression patterns in co-expression modules identified by weighted gene co-expression network analysis (WGCNA).


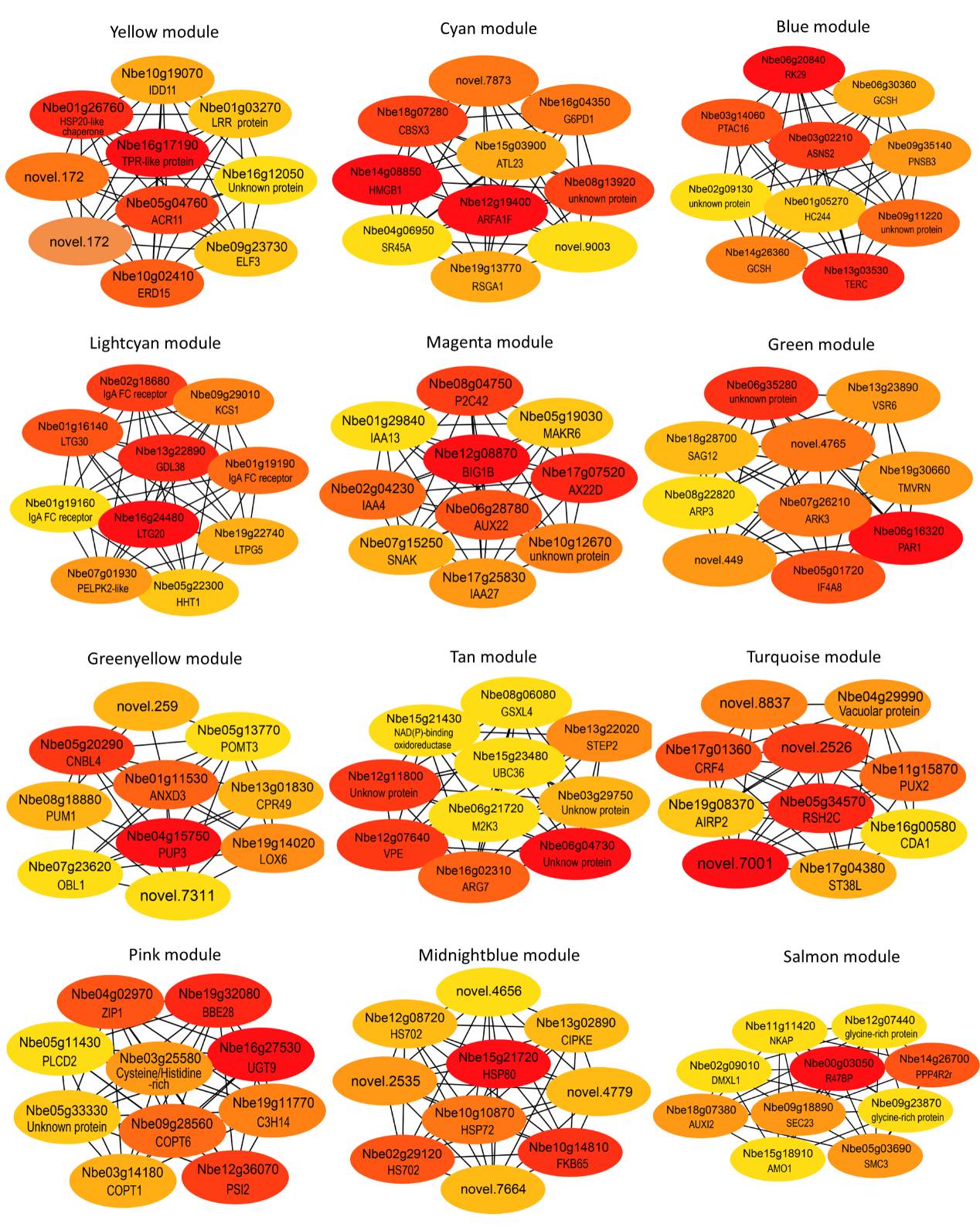


**Figure S5** Regulatory networks of the top 10 hub genes in each module.


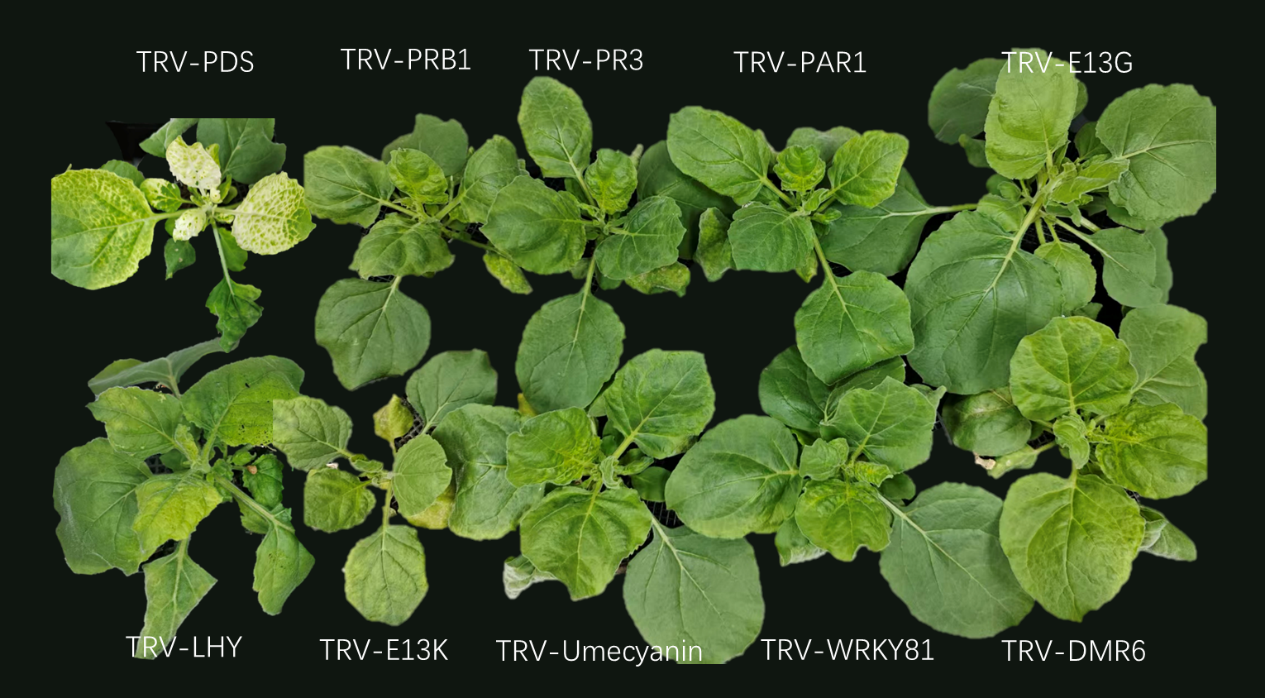


**Figure S6** Phenotypes of VIGSed plants. Photographs were taken at 10 dpi withTRV.


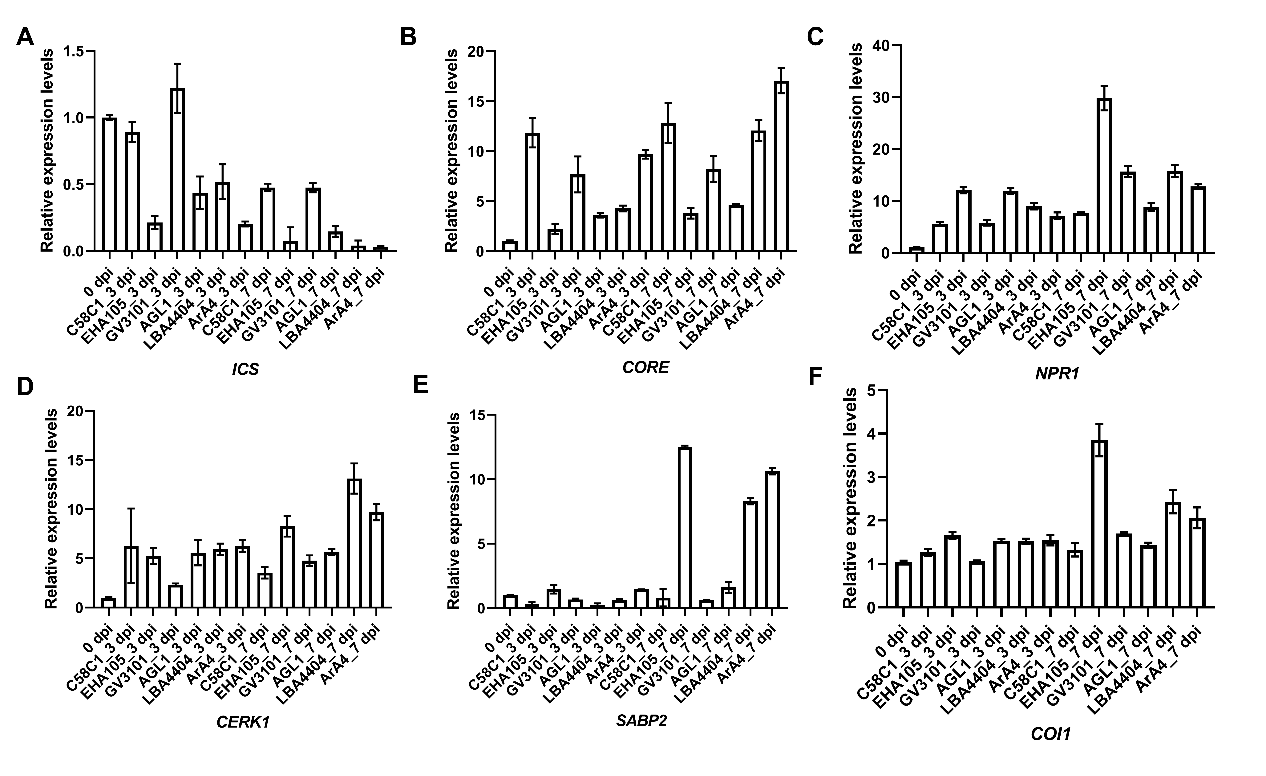


**Figure S7** qPCR validation of transcriptome data by detecting immune-related genes modulating recombinant protein expression.
